# Effects of iron repletion on brain iron content, myelination, neural network activation, and cognition

**DOI:** 10.1172/jci.insight.194442

**Published:** 2025-10-21

**Authors:** Eldad A. Hod, Christian Habeck, Hangwei Zhuang, Alexey Dimov, Pascal Spincemaille, Debra Kessler, Zachary C. Bitan, Yona Feit, Daysha Fliginger, Elizabeth F. Stone, David Roh, Lisa Eisler, Stephen Dashnaw, Elise Caccappolo, Donald J. McMahon, Yaakov Stern, Yi Wang, Steven L. Spitalnik, Gary M. Brittenham

**Affiliations:** 1 Department of Pathology and Cell Biology,and; 2 Cognitive Neuroscience Division in Neurology, Columbia University College of Physicians and Surgeons, New York Presbyterian Hospital, New York, New York, USA.; 3 Department of Radiology, Weill Cornell Medical College, New York, New York, USA.; 4 New York Blood Center, New York, New York, USA.; 5 Department of Neurology,; 6 Department of Pediatrics,; 7 Department of Anesthesiology,; 8 Department of Radiology, and; 9 Department of Medicine, Columbia University College of Physicians and Surgeons, New York Presbyterian Hospital, New York, New York, USA.

**Keywords:** Hematology, Neuroscience, Neuroimaging

## Abstract

**BACKGROUND:**

Blood donation increases the risk of iron deficiency, but its effect on brain iron, myelination, and neurocognition remains unclear.

**METHODS:**

This ancillary study enrolled 67 iron-deficient blood donors, 19–73 years of age, participating in a double-blind, randomized trial. After donating blood, positive and negative susceptibility were measured using quantitative susceptibility mapping (QSM) MRI to estimate brain iron and myelin levels, respectively. Furthermore, neurocognitive function was evaluated using the NIH Toolbox, and neural network activation patterns were assessed during neurocognitive tasks using functional MRI (fMRI). Donors were randomized to i.v. iron repletion (1 g iron) or placebo, and outcome measures repeated approximately 4 months later.

**RESULTS:**

Iron repletion corrected systemic iron deficiency and led to trends toward increased whole brain iron (*P* = 0.04) and myelination (*P* = 0.02), with no change in the placebo group. Although overall cognitive performance did not differ significantly between groups, iron-treated participants showed improved engagement of functional neural networks (e.g., memory pattern activation during speed tasks, *P* < 0.001). Brain region-specific changes in iron and myelin correlated with cognitive performance: iron in the putamen correlated with working memory scores (*P* < 0.01), and thalamic myelination correlated with attention and inhibitory control (*P* < 0.01).

**CONCLUSION:**

Iron repletion in iron-deficient blood donors may influence brain iron, myelination, and function, with region-specific changes in iron and myelination linked to distinct cognitive domains.

**REGISTRATION:**

ClinicalTrials.gov NCT02990559

**FUNDING:**

This work was funded by the NIH.

## Abstract 2

Blood donors with iron deficiency may benefit from iron treatment, which appears to affect brain function, brain iron and myelin levels.

## Graphical abstract

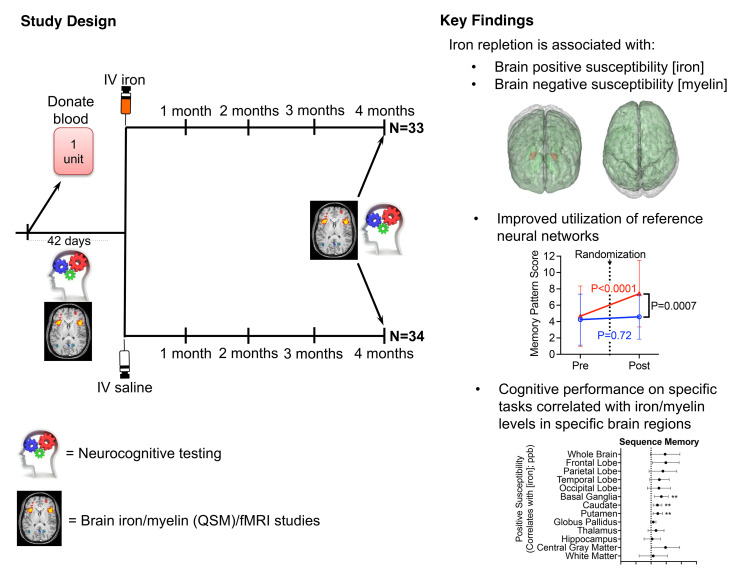

## Introduction

Iron is an essential trace element critical for the proper functioning of virtually all biological systems, including oxygen transport, energy metabolism, immunity, and cognition (1). Over 118 million units of blood are donated worldwide each year, most coming from altruistic volunteer repeat donors (2, 3). One consequence of blood donation is iron deficiency, and blood collection organizations currently face challenging decisions regarding how and/or whether to prevent or treat iron deficiency in their donors (4). Our recently completed Donor Iron Deficiency Study (DIDS) found that current regulatory criteria for blood donation maintain red cell storage quality for transfusion without exposing adult donors to measurable adverse effects on quality of life or cognition resulting from blood donation-induced iron deficiency (5, 6). Nonetheless, the effect of iron repletion on iron and myelin levels in specific brain regions of interest, and correlations of these levels with cognitive performance in these same donors, were not reported.

Iron acquisition by the brain is tightly controlled in an age-related and brain region–dependent process (7, 8). Iron deficiency in infancy is associated with poorer neurocognition (9), but the consequences of blood donation-induced iron deficiency in adults have not been rigorously examined. Iron is a key component of systems in the brain that are responsible for myelination and dopamine synthesis (7). Although most myelination is thought to occur postnatally and through adolescence, it continues throughout adult life (10, 11). Myelination increases conduction velocity across axons, and active central myelination is important for learning (12–14). Understanding the influence of iron repletion on brain iron and myelination is vital, as it provides insights into the potential mechanisms underlying the cognitive impairments observed in individuals with iron deficiency.

In our previously reported parent DIDS trial (5, 6), healthy blood donors who met donation standards but were iron deficient by laboratory criteria (i.e., ferritin < 15 μg/L and zinc protoporphyrin > 60 μMol/mol heme) were prospectively randomized to receive iron (1 g of low molecular weight iron-dextran i.v.) or placebo (i.v. saline) in a double-blind fashion. We now report on neurocognitive testing and magnetic resonance imaging (MRI) studies performed before and 3–5 months following randomization (with a target of 4 months, corresponding to the lifespan of RBCs, to allow complete replacement of the erythroid compartment under the iron conditions established by randomization). We used quantitative susceptibility mapping (QSM) MRI studies with magnetic susceptibility source separation to estimate brain iron and myelination (8, 15, 16). We administered selected instruments from the NIH Toolbox, a computerized battery of tests designed for clinical research across the lifespan, to measure neurocognitive function. We used functional MRI (fMRI) to assess utilization of reference neural network activation patterns during neurocognitive tasks. Here, we show that: (a) iron repletion improved fMRI measures of neural network activation patterns in the brain, (b) iron repletion increased estimates of iron and myelin levels in specific brain regions, and (c) changes in performance in specific neurocognitive tasks correlated with changes in estimates of iron and myelin in specific brain regions.

## Results

### Study participants.

**Figure 1:**
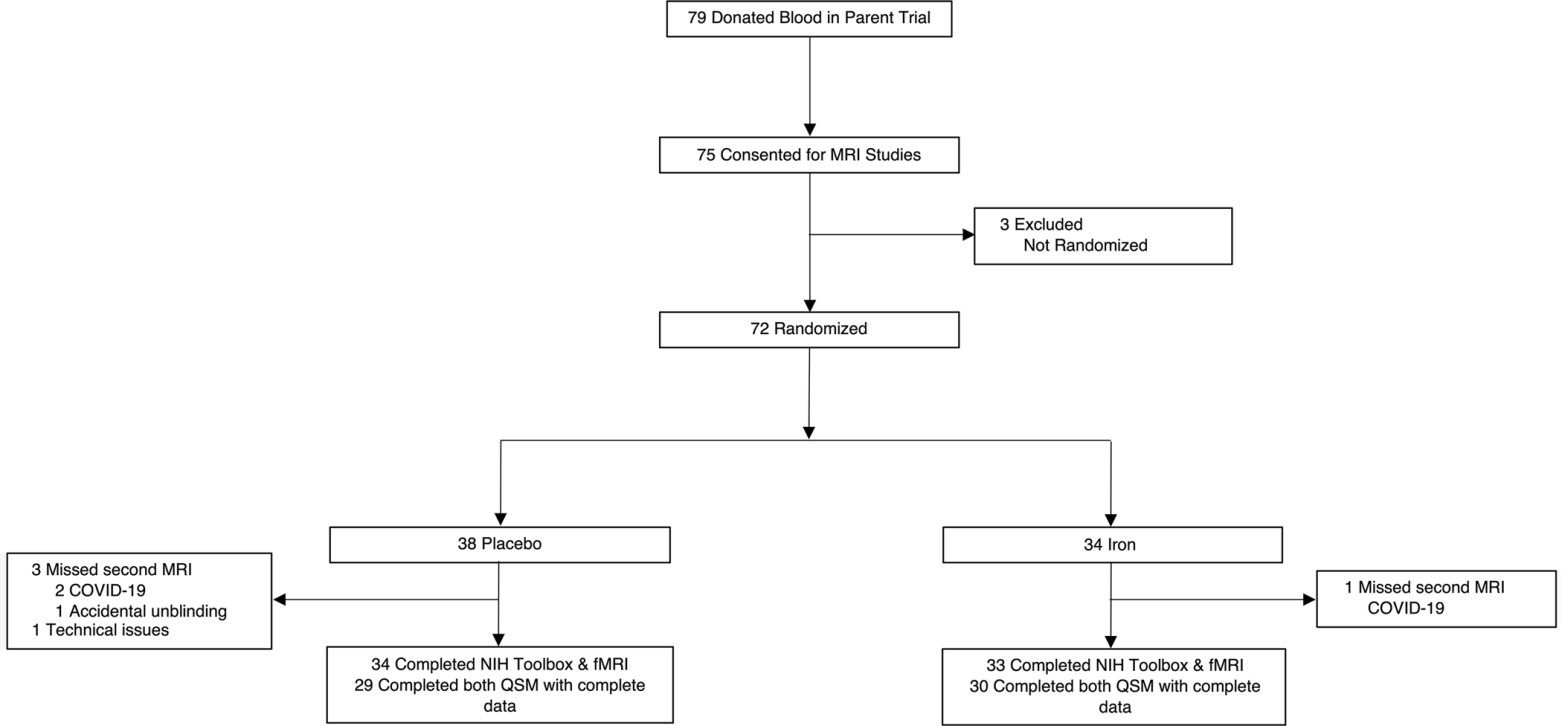
Study flow diagram. CONSORT flowchart showing recruitment, participation, and completeness of main outcomes. fMRI, functional MRI; QSM, quantitative susceptibility mapping.

Of 79 subjects who donated blood in the parent trial, 75 consented to ancillary neuroimaging studies. Seventy-two of the latter were successfully randomized in the parent trial, with 34 assigned to iron repletion and 38 to placebo (Figure 1). Due to technical issues, only 30 subjects in the iron repletion group and 29 subjects in the placebo group successfully completed both scheduled QSM studies and were included in the analyses of brain iron and myelin estimates.

The baseline demographic data in Table 1 show no significant between-group differences for the iron repletion and placebo groups. Nonetheless, as previously reported in the primary outcome report from the parent trial, laboratory measures of systemic iron status (e.g., hemoglobin and ferritin) were both significantly increased by randomization to iron repletion (Table 1). The median time from randomization to the second neuroimaging study did not differ between placebo and iron repletion groups (127 [IQR: 106–138] versus 119 [113–143] days; *P* = 0.99).

### Cognitive performance outcomes.

**Figure 2:**
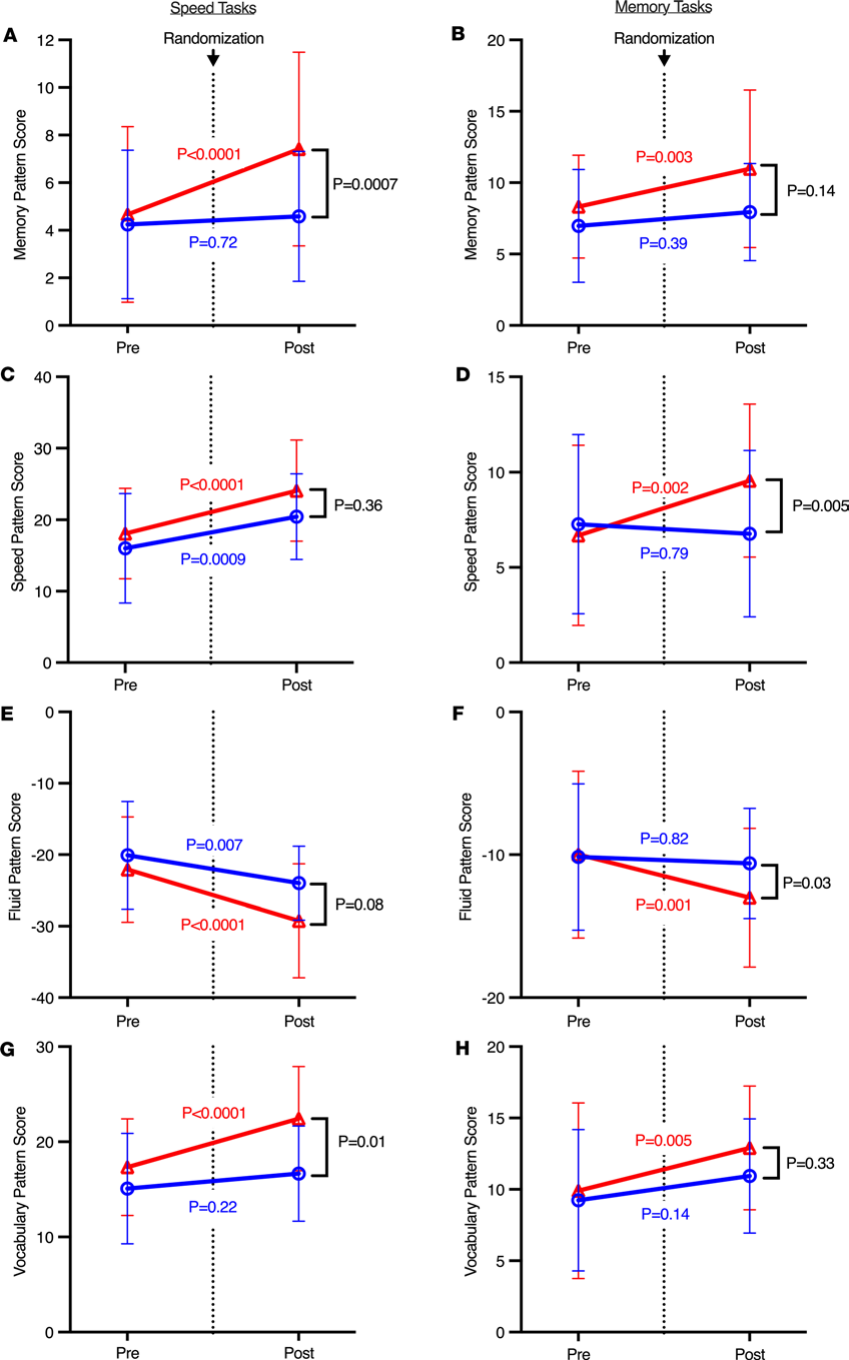
Mean spatial covariance pattern scores on speed and memory tasks performed during fMRI before and after randomization. Mean spatial covariance pattern scores for 4 prespecified reference ability neural networks (RANNs) during performance of speed and memory tasks in fMRI testing are shown by treatment group from before and after randomization. These pattern scores reflect the similarity of each participant’s fMRI activation pattern while performing either speed or memory tasks (left and right column, respectively) to validated RANNs associated with specific cognitive domains (i.e., memory, speed, fluid, and vocabulary; in rows, as labeled). Data are shown as mean ± SD. Placebo in blue and iron repletion group in red with colored *P* value representing the adjusted *P* value from a Šídák’s multiple-comparison test of the within group difference from before to after randomization score in a mixed model analysis. *P* value in black represents the significance of the mixed model interaction term between treatment group and time point. Additional fixed effects include the interaction between sex and age. The subject intercept was included in the model as a random effect. An unstructured covariance matrix was used to model the within-subject variance-covariance errors. (**A**–**H**) Mean RANN pattern scores for memory (**A** and **B**), speed (**C** and **D**), fluid cognition (**E** and **F**), vocabulary (**G** and **H**) are shown for the 3 tests of processing speed (left) and memory (right).

Cognitive performance was assessed using the NIH Toolbox administered in a standardized setting on a tablet device and using 6 selected tests, 3 assessing processing speed and 3 assessing memory administered during the fMRI procedure. Median scores for each component measure did not significantly differ between groups at any time before or after randomization (Supplemental Figures 1 and 2; supplemental material available online with this article; https://doi.org/10.1172/jci.insight.194442DS1). Nonetheless, median neural network activation pattern scores for memory and vocabulary, and for processing speed and fluid cognition on Reference Ability Neural Networks (RANNs) (17, 18), were significantly affected by iron repletion during performance on the speed- or memory-related tasks, respectively (Figure 2). These pattern scores quantify how closely an individual’s fMRI activation aligns with validated RANNs linked to specific cognitive domains (i.e., memory, processing speed, fluid reasoning, and vocabulary) during performance of speed or memory tasks.

### Effect of iron repletion on brain iron and myelin levels.

**Figure 3:**
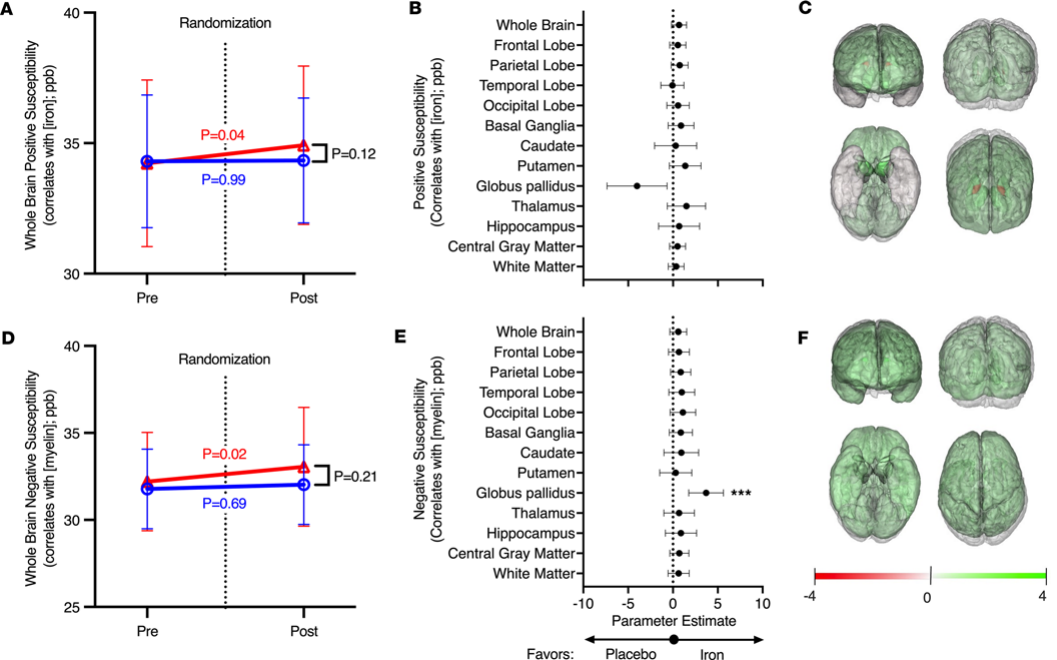
Effect of iron repletion on iron and myelin levels in brain regions of interest. Iron and myelin levels in specific brain regions were estimated by QSM in all subjects, before and after randomization to iron repletion. (**A** and **D**) Mean whole iron (**A**) and myelin (**D**) levels are shown by treatment group from before to after randomization. The data are shown as mean ± SD. Placebo in blue and iron repletion group in red with colored *P* value representing the adjusted *P* value from a Šídák’s multiple-comparison test of the within-group difference from pre- to postrandomization score in a mixed model analysis. *P* value in black represents the significance of the mixed model interaction term between treatment group and time point. Additional fixed effects include sex, age, and the interaction between sex and age. The subject was included in the model as a random effect. An unstructured covariance matrix was used to model the within-subject variance-covariance errors. (**B** and **E**) Forest plots for the parameter estimate (95% CIs) for the effect of randomization (i.e., interaction term between treatment group and time point) for iron (**B**) and myelin (**E**) levels in specific brain regions of interest. (**C** and **F**) Summary heatmap of these parameter estimates for iron (**C**) and myelin (**F**) levels on a brain atlas. Parameter estimates converted to corresponding color intensity from red to green as shown in the legend. ***P < 0.001.

Iron concentration in the whole brain, estimated by positive susceptibility in QSM MRI, did not differ significantly when comparing the iron repletion to the placebo group (Figure 3A). Nonetheless, in the within-group comparison, whole brain iron increased in the iron repletion (*P* = 0.04) but not in the placebo (*P* = 0.99) group after randomization. A forest plot (Figure 3B) of the interaction term between the time point (i.e., before or after randomization) and the randomization group (i.e., iron repletion or placebo), is shown for the mixed model examining the relationship between iron in a particular region of interest in the brain, while adjusting for age, sex, and the interaction between age and sex. The results favored iron repletion (i.e., showed a positive association) for all brain regions examined except the globus pallidus and temporal lobe. A heatmap of the strength of the association between brain regions and randomization to iron repletion highlights the trend to increased iron in most brain regions (Figure 3C).

Myelin concentration in the whole brain, estimated by negative susceptibility in QSM MRI, did not differ significantly when comparing the iron repletion to placebo group (Figure 3D). In the within-group comparison, iron repletion increased whole brain negative susceptibility (*P* = 0.02), but placebo had no significant effect (*P* = 0.69). A forest plot (Figure 3E) of the interaction term between the time point and the randomization group in the mixed model examining the relationship between negative susceptibility in a particular region of interest in the brain, favored iron repletion for all brain regions examined, and reached statistical significance for the globus pallidus (*P* = 0.0003). A heatmap of the strength of the association between brain regions and randomization to iron repletion highlights the general trend to increased negative susceptibility (correlating with myelin levels) in the brain following iron repletion (Figure 3F).

### Iron and myelin levels in particular regions of interest are associated with cognitive performance on specific tasks.

Excluding randomization from the mixed effects models altogether, while still accounting for the repeated measures for each subjects and adjusting for age, sex, and the interaction between sex and age, we next examined the relationship between iron parameters in blood samples (e.g., hemoglobin, ferritin, soluble transferrin receptor, and hepcidin; Supplemental Figure 3) and the cognitive test scores using the NIH Toolbox. No blood parameter results were significantly associated with these cognitive scores.

**Figure 4:**
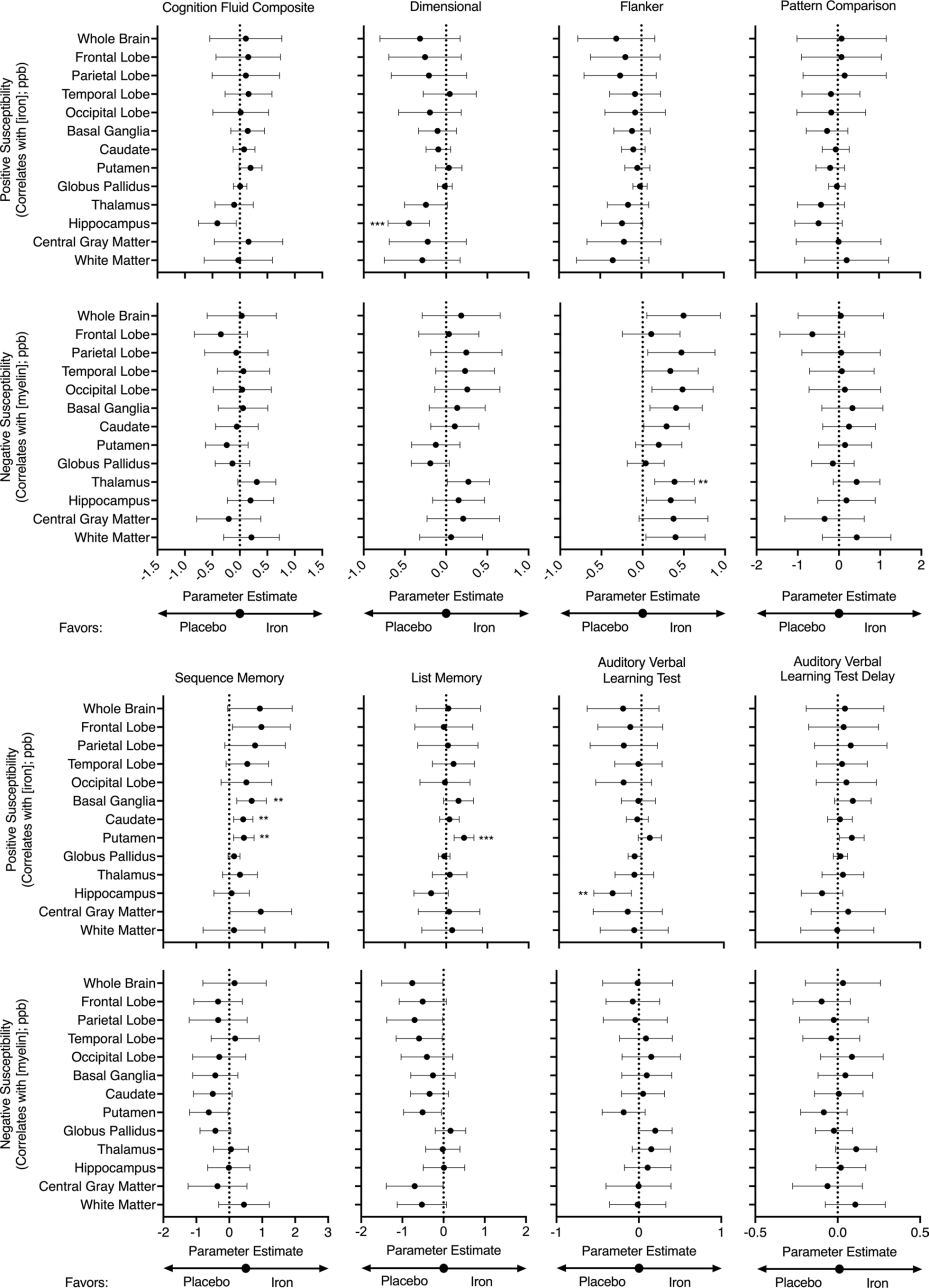
Relationship between brain iron and myelin levels and cognitive performance scores. Positive and negative susceptibility, which correlate with iron and myelin levels, respectively, were measured in all subjects before and after randomization to iron repletion by QSM. In separate mixed effect models for iron and myelin levels in a specific region of interest as labeled, and accounting for the repeated measures and the time point, the parameter estimate (β) and 95% CI is presented in a forest plot for each cognitive test performed using the NIH Toolbox standardized scores. Additional fixed effects include sex, age, and the interaction between sex and age. The subject was included in the model as a random effect. A variance component covariance matrix was used to model the within-subject variance-covariance errors. Larger positive parameter estimates represent stronger correlations between iron or myelin concentration in a particular brain region of interest and cognitive test performance on a specific task, as labeled. **P < 0.01, ***P < 0.001.

Finally, we examined relationships between cognitive scores and positive and negative susceptibility (i.e., estimates of iron and myelin levels, respectively) in specific brain regions of interest (Figure 4). Iron levels in the hippocampus were negatively associated with the NIH Toolbox measure of attention and cognitive flexibility (Dimensional Change Card Sort Test scores [β estimate of –0.45; *P* < 0.001]) and the measure of immediate memory and verbal learning (Auditory Verbal Learning Test [Rey] scores [β estimate of –0.35; *P* < 0.01]). Iron levels in all brain regions examined were positively associated with the NIH Toolbox measure of episodic memory (Picture Sequence Memory Test scores, with parameter estimates for the basal ganglia, caudate, and putamen showing the strongest associations [β estimates of 0.68, 0.43, and 0.44, respectively; all *P* < 0.01]). Iron levels in the putamen were also positively associated with the NIH Toolbox measure of working memory (List Sorting Working Memory Test scores [β estimate of 0.43; *P* < 0.001]). Myelin levels (as measured by negative susceptibility) in all brain regions examined were positively associated with the NIH Toolbox measure of attention and inhibitory control (Flanker Inhibitory Control and Attention Test scores, with the parameter estimate for the thalamus showing the most significant association [β estimate of 0.39; *P* < 0.01]).

## Discussion

This neurocognitive and neuroimaging ancillary study to the DIDS randomized, double-blind, placebo-controlled trial identified 3 major results from our study of iron-deficient blood donors. First, i.v. iron repletion was associated with altered utilization of reference neural network activation patterns for memory and vocabulary and for processing speed and fluid cognition (Figure 2) (19). Second, although most regional effects did not reach statistical significance, iron repletion was consistently associated with increases in both positive and negative susceptibility (i.e., estimates of iron and myelin levels, respectively) across nearly all brain regions. This uniform directionality lends support to a potential biological effect, as purely random variation would be expected to produce estimates distributed on both sides of zero (Figure 3). Third, in specific brain regions, changes in positive and negative susceptibility correlated with changes in performance on NIH Toolbox measures of attention and cognitive flexibility, immediate memory and verbal learning, episodic memory, working memory, and attention and inhibitory control (respectively, the Dimensional Change Card Sort, Auditory Verbal Learning, Picture Sequence Memory, List Sorting Working Memory, and Flanker Inhibitory Control and Attention tests; Figure 4). In particular, in the putamen, which regulates movement and various types of learning, an increase in iron from before to after randomization was strongly associated with improvements in working and episodic memory (i.e., List Sorting Working Memory and Picture Sequence Memory tests). Altogether, these results provide evidence that iron repletion (1 g low–molecular weight iron dextran i.v.) of iron-deficient blood donors (i.e., ferritin < 15 ng/mL) improved measures of neural network activation patterns in the brain. Furthermore, the results suggest an effect of iron repletion on increased iron and myelin levels in specific brain regions. Finally, performance in specific neurocognitive tasks correlated with positive and negative susceptibility (correlating with iron and myelin, respectively) in specific brain regions.

In contrast, changes in cognitive performance were not associated with changes in systemic measures of iron status, such as blood levels of hemoglobin, hepcidin, soluble transferrin receptor, or ferritin (Supplemental Figure 3). Taken together, these findings suggest that QSM-derived positive and negative susceptibility measurements (reflecting iron and myelin levels, respectively) in specific regions of interest capture neurobiological processes that are more closely aligned with cognitive performance than traditional blood-based measures. While such neuroimaging approaches are unlikely to serve as practical tools for monitoring cognition in clinical settings, they provide important mechanistic insight into how iron status may influence brain function. Nonetheless, despite the effect of randomization on brain iron and myelin levels, along with correlations between changes in brain iron and myelin in specific regions of interest and cognitive performance on specific tasks, the intention-to-treat analysis for the effect of randomization to iron repletion on cognitive performance did not result in significant differences. This might be due to our limited sample size, given the heterogeneity of donor ages and the variability in cognitive performance represented in our sample.

Iron is a cofactor in several neuron-specific functions, such as myelin and neurotransmitter (e.g., dopamine) synthesis (20). Although some effects of iron deficiency during infancy may be irreversible (9), in animal studies, the iron-deficient brain rapidly regains iron following systemic iron repletion (21, 22). For example, in adult rats, supplementation with isotopically enriched iron increases iron incorporation into the brain (23), suggesting that iron is in dynamic flux even in fully developed adult brains. Iron is heterogeneously distributed in the brain, with the highest concentrations in the substantia nigra, putamen, globus pallidus, deep cerebellar nuclei, red nucleus, nucleus accumbens, and hippocampus (24, 25). Although these iron levels are age and sex dependent (8), with increased iron associated with neurodegeneration in the aging brain (26), not all brain regions are equally sensitive to changes in body iron status (27). For example, dopaminergic tracts are highly sensitive to diminished brain iron (27–32). Iron deficiency is also associated with increases in dopamine levels and decreases in striatal dopamine D_2_ receptor density (33). In addition, there is a strong association between striatal D_2_ receptor binding and cognitive performance (34). Importantly, extracellular striatal dopamine levels return to normal after iron repletion (28). Furthermore, iron concentration increases throughout adolescence, with some of the greatest increases occurring in the putamen; iron concentration in the putamen is also positively associated with overall cognitive performance in this age group (35).

In our study, iron repletion was associated with positive trends in positive susceptibility (i.e., iron concentrations) across nearly all brain regions of interest, with the notable exceptions of the globus pallidus and temporal lobe. The globus pallidus is unique in that it does not exhibit a consistent increase in positive susceptibility (i.e., iron) across the lifespan and seems to plateau in adulthood (8, 25). Iron is stored in oligodendrocytes (25), and many subcortical gray matter structures contain or border deep white matter (36). In neurodegenerative conditions, myelin loss is associated with focal iron accumulation (37), and reduced dietary iron has been associated with impaired myelin production (20, 38). Against this background, our observation of a significant iron repletion effect in the globus pallidus, characterized by increased negative susceptibility (interpreted as myelin-related) together with decreased positive susceptibility (interpreted as iron-related), may suggest a shift toward utilization of iron for myelin production. However, this interpretation should be made cautiously. This result may also reflect methodological limitations of interpreting positive and negative susceptibility strictly as markers of iron and myelin, respectively, and/or unique structural features of the globus pallidus that distinguish it from other regions. Notably, in all other regions examined, we observed concordant (though nonsignificant) increases in both positive and negative susceptibility with iron repletion.

Increased iron in the putamen was positively and most strongly associated with performance on List and Sequence Memory tests, while increased iron in the hippocampus was negatively associated with performance on the Dimensional Card Sorting and Auditory Verbal Learning tests. Because QSM-positive susceptibility correlates with storage iron (8, 15), it is possible that a decrease in positive susceptibility may be associated with mobilization of iron toward cellular functions, such as myelin formation, in which case negative susceptibility would increase. This is consistent with finding that randomization was associated with increased negative susceptibility (i.e., myelination) in all areas examined. Indeed, due to the high metabolic demand of myelination, oligodendrocytes are the cells with the highest level of iron in the brain, and iron availability is critical in the remyelination process following a demyelinating insult (39).

Axon myelination increases the speed of impulse propagation, but it also has emerged as an important component of plasticity, memory, and learning (40, 41). In particular, and as strongly observed in this DIDS ancillary study (Figure 4), intracortical myelin levels correlate with differences in intraindividual performance during attentional tasks (e.g., Flanker Inhibitory Control and Attention test) in adults (41). Other longitudinal studies also support our finding that myelin levels correlate with cognitive performance in cognitively unimpaired adults (42). In addition, myelin levels increase as children age into adulthood; interestingly, myelination, as assessed by T1w/T2w ratio, inversely correlated with performance on NIH Toolbox cognitive measures in children and adolescents (43). Nonetheless, our study did not include children and adolescents, and the effect may differ in different stages of neurodevelopment.

There is strong age and sex dependence in iron and myelin levels in the brain. Thus, one strength of our study is that each subject was their own control (i.e., repeated measures), with longitudinal measures taken once before and once after randomization to i.v. iron repletion. Double blinding was also key in preventing any potential treatment biases. Nevertheless, one limitation of this study is that we only assessed cognitive performance and iron and myelin content in brain structures once at ~120 days following randomization. Because iron’s effects on performance may take longer, or may manifest more acutely following repletion, future studies would benefit from additional timed measurements to identify more precisely the timing of the effects of iron repletion. It is also possible that other, more sensitive, measures of cognitive performance, not utilized in this study, would be affected more robustly by iron repletion. Another limitation of our study is the use of QSM-negative susceptibility as an estimate of myelin levels. While QSM has theoretical appeal because it probes the intrinsic electronic properties of the myelin sheath, its application to myelin quantification is methodologically challenging. More well-established approaches for estimating myelin content include myelin water imaging (MWI) and quantitative R1 mapping (44–46), which infer myelin content from water trapped between myelin layers or from the surrounding free-water environment. These relaxation-based techniques are more widely validated and accepted, although they are indirect measures. Our interpretation of QSM-negative susceptibility as myelin is complicated by several factors. For example, negative susceptibility is not a pure marker of myelin, but it can also be influenced by orientation anisotropy, partial volume effects, and contributions from nonmyelin macromolecules. Susceptibility values also highly depend on the choice of reference tissue, reconstruction algorithms, and regularization parameters, which can introduce systematic variability across studies and implementations. In our study, cerebrospinal fluid (CSF) was used as the zero reference; however, this assumes that CSF susceptibility remains unchanged by iron repletion, which may not strictly hold and could introduce bias in the interpretation of whole-brain effects. Due to the acquisition time constraints, the voxels in the acquired gradient echo data were anisotropic in size. Voxel anisotropy reduces phase contrast in the acquired data, causing systematic underestimation, blurring, and streaking artifacts, particularly along the low-resolution axis (47, 48). Additionally, subcortical regions (e.g., putamen, thalamus, globus pallidus) contain complex mixtures of iron- and myelin-rich tissue, making it difficult to disentangle the relative contributions of these sources to the measured signal. Finally, susceptibility source separation itself is still an evolving field with no consensus gold standard, and QSM cannot yet be considered a definitive tool for quantifying myelin. For these reasons, caution is warranted in interpreting negative susceptibility in our study as a direct measure of myelin. Future cross-validation against established myelin-sensitive imaging techniques will be essential to validate and extend our findings further.

There is a “healthy donor effect” in that blood donors are healthier than nonblood donors (49), a limitation that could have been exacerbated by including only blood donors who could commit significant time for the multiple visits that the DIDS trial required. Thus, our findings should not be generalized to individuals with typical nutritional iron deficiency. Furthermore, although we used a more stringent α level of 0.01 to reduce the risk of type I errors, we did not apply multiple comparison adjustments for secondary or exploratory endpoints. This limitation increases the possibility of false positives; therefore, the findings should be interpreted with caution. Nevertheless, the consistent patterns observed (e.g., positive susceptibility in the putamen correlating with both independently administered memory-based tests; negative susceptibility across regions of interest associating with Flanker Inhibitory Control and Attention Test scores; Figure 4) support the likelihood that these associations reflect meaningful biological effects rather than chance findings. Importantly, iron repletion also had a strong effect on the utilization of RANNs, which are validated neural activation patterns known to capture much of the variance in age-related cognitive change (18, 19). This finding provides further evidence that iron status influences core neural systems underlying cognition, highlighting a biologically significant effect of iron repletion beyond traditional behavioral or blood-based measures. Finally, because of the complex relationship between age and brain iron/myelin levels, although beneficial for generalizability, the heterogeneity of ages represented in our study, ranging from 19 to 73 years old, may also have introduced additional variability in our outcomes, limiting statistical power. Larger studies in more defined age populations are necessary for fully elucidating the relationships between donation-induced iron deficiency and cognitive performance.

In conclusion, in the DIDS neurocognitive ancillary study, iron repletion of iron-deficient blood donors increased QSM-positive and -negative susceptibility, which are consistent with increases in brain iron and myelin concentrations. Furthermore, iron repletion was associated with improved utilization of reference neural network activation patterns on previously demonstrated RANNs that correlate with cognition (17, 18). Nonetheless, these changes did not improve overall cognitive performance as measured by NIH Toolbox testing. Thus, these findings do not support changing current regulations directed at reducing iron deficiency to improve the safety of altruistic, volunteer blood donation. Further studies are necessary to determine the full effect of donation-induced iron deficiency on brain biochemistry throughout the lifespan — and especially for brain development and cognition in younger teenage donors, who comprise 10%–20% of blood donors (2, 50, 51) but who were not included in this study.

## Methods

### Sex as a biological variable.

Study enrollment was not restricted by sex. Due to the higher prevalence of iron deficiency among females, more female subjects were enrolled in the parent study (6), resulting in their overrepresentation in this ancillary study. However, sex, and the interaction between age and sex, were included in the mixed effects models examining iron and myelin levels in the brain.

### Trial design and participants.

This was an ancillary, observational study linked to the previously described parent DIDS trial (5, 6). In the parent trial, nonanemic (i.e., hematocrit: male > 39%; female > 38%), blood donors (male ≥ 2 and female ≥ 1 whole blood donations in the past year) with defined laboratory evidence of iron deficiency (i.e., ferritin < 15 ng/mL and zinc protoporphyrin > 60 μMol/mol heme) were recruited from the New York Blood Center in New York, New York, USA. Subjects donated a standard whole blood unit and then were randomized to iron repletion (1 g low–molecular weight iron dextran i.v.) or placebo (saline i.v.) in double-blind fashion. All subjects from the parent trial were eligible for this ancillary study, except those in whom MRI was contraindicated because of non-MRI-safe metals. MRI sequences included QSM and fMRI. MRI of the brain was performed first after a standard whole blood donation and before randomization, and then 120 ± 30 days following randomization. All subjects signed a separate informed consent for the MRI studies. The parent trial consent form included the NIH Toolbox neurocognitive testing and laboratory testing that were also performed at 4 prespecified time points. For the NIH Toolbox and laboratory measures, only the 2 time points after and most proximal to each MRI study were considered (Supplemental Figure 1). Scheduling and logistic communications were performed by blinded study coordinators. Furthermore, all laboratory testing and outcome measures were evaluated by blinded individuals.

### Procedures.

Donors were recruited between January 17, 2017, and January 29, 2021, with final 5-month follow-up completed by October 5, 2021. The NIH Toolbox neurocognitive tasks were previously described (6). During fMRI data acquisition, each subject performed 6 computerized tasks, 3 for assessing perceptual/processing speed and 3 assessing episodic memory (17, 18). Task administration and data collection were controlled by a PC computer running E-Prime v2.08 software, and electronically synchronized with the MR scanner. Task stimuli were back-projected onto a screen located at the foot of the MRI bed by an LCD projector. Subjects viewed the screen via a mirror system located in the head coil. Task responses were made on a LUMItouch response system (Photon Control Company) and behavioral response data were recorded on the task computer. Prior to the scan session, computerized training was administered for the tasks to be administered. To assess perceptual/processing speed, the Digit Symbol, Letter Comparison, and Pattern Comparison tests were administered. To assess episodic memory, the Logical Memory, Word Order Recognition, and Paired Associates tasks were administered (see Supplemental Methods for more details).

### MRI procedures.

For all subjects, MRI was performed twice: once prior to randomization and once after randomization. Scans were acquired on either a 3.0T Philips Achieva or a 3T General Electric Signa scanner, with each participant scanned on the same scanner at both time points to ensure consistency. MRI sequences comprised: (a) a scout, T1-weighted imaging acquired to determine subject position, (b) an MPRAGE imaging for structural analysis, (c) EPI imaging for the functional imaging, and (d) a 3-dimensional flow-compensated gradient echo sequence with scan time of 6.5 minutes for QSM (8, 15, 16). The pulse sequence parameters are in Supplemental Table 1 and additional details are provided in the Supplemental Methods. Representative QSM imaging examples are provided in Supplemental Figure 4. A neuroradiologist reviewed each scan to confirm the absence of clinically significant findings.

### Outcomes.

The primary outcome measure was the within-subject change in QSM whole brain–positive magnetic susceptibility (15, 16), that occurred between the first scan, under conditions of iron deficiency, and the second scan, after randomization to iron repletion or placebo. Secondary outcomes included the within-subject change in QSM-negative magnetic susceptibility (15, 16), in the following prespecified regions of interest: frontal lobes, parietal lobes, temporal lobes, occipital lobes, basal ganglia, caudate, putamen, globus pallidus, thalamus, hippocampus, central gray matter, and white matter.

The RANN Study (18) utilized the identical fMRI tasks to derive previously demonstrated RANNs (17, 18) underlying 4 reference abilities: memory, speed, fluid cognition, and vocabulary. A neural network activation pattern score derived from using each reference ability was determined for each task, as described in the Supplemental Methods (19). Median neural network activation pattern scores for the 3 processing speed and the 3 memory tasks were utilized in the final analysis. Of the super set of 67 participants whose task activation data for 6 tasks were analyzed for this paper, 51 had their fMRI data acquired on a 3T Philips Achieva scanner, while 16 had their fMRI data acquired on a 3T General Electric Signa scanner. The scanner effect was purely between subjects, and there were no within-participant time point differences in scanner assignment. We applied the MATLAB version of the correction technique ComBat (52–54) to each of the 6 activation tasks considered, without any covariate array. Scanner assignment was not confounded with treatment randomization (*P* = 0.62).

### Brain atlas heat map.

Three-dimensional brain images were formed using Mango version 4.1 (Research Imaging Institute) software. The International Consortium for Brain Mapping (ICBM) (55) 452 T1 brain atlas was used as the template. Parameter estimates from the mixed models representing the strength of the association between the change in iron or myelin concentration and randomization to iron repletion or placebo were normalized and were used to create a color scale, with green representing positive values, red for negative, and white for neutral.

### Statistics.

The primary null hypothesis was that there would be no significant between-group difference in the mean within-subject change in whole brain–positive magnetic susceptibility (i.e., whole brain iron) from the first study under conditions of iron deficiency and the second study after randomization to iron repletion or placebo. The primary null hypothesis and all secondary outcomes were tested using linear mixed models for repeated measures to compare differences in the iron repletion and placebo group temporal course at the 2 defined time points when adjusting for sex, age, and the interaction between sex and age. In additional tertiary studies, linear mixed models for iron and myelin levels in each brain region, or blood iron measure, were generated to quantify the relationship between that parameter to the cognitive score at each time point. The time point, sex, age, and interaction between sex and age were all included in the model, but randomization assignment was excluded. Subjects needed to complete both MRI study outcome measures to be included in these ancillary study analyses. This study was limited in sample size to that of the parent trial. The test-retest reproducibility of QSM on the same individual using a 3T MRI scanner yields a standard deviation of approximately 3.2 ppb (56). With 30 evaluable subjects in each trial group, and assuming a 1 SD difference in brain iron in a particular nucleus with iron repletion, we expected to have 90% power (α = 0.01). For context, average iron concentrations in the caudate and putamen are approximately 89.2 and 88.0 ppb, respectively (8), making a 3.2 ppb difference equivalent to less than a 4% change; this is well within the sensitivity range of our imaging protocol to detect physiologically meaningful variation. We used *P* < 0.01 to determine significance and made no multiple comparison adjustments for the secondary and tertiary end points. SAS Studio version 3.8 (SAS Institute Inc.) was used for all analyses and Prism version 9.3 (GraphPad) was used for making figures.

### Study approval.

The institutional ethics board at Columbia University Irving Medical Center approved the research protocol.

### Data availability.

Deidentified individual participant data used in this study are available in the Supporting Data Values file. Additional data may be obtained from the corresponding authors upon reasonable request. Researchers interested in accessing these materials may contact the corresponding authors for further information.

## Author contributions

EAH, GMB, EC, CH, YS, DJM, DR, and SLS designed the study and analyzed the results. DK, ZCB, YF, DF, LE, EFS, and SD helped coordinate many crucial aspects of the trial to ensure its safe and successful operational execution. ZCB, YF, DF, and LE enrolled subjects, collected data, and helped interpret results. HZ, AD, PS, and YW analyzed the quantitative susceptibility mapping data. EC, CH, and YS designed and helped interpret the cognitive performance outcomes. EAH and DJM performed the statistical analyses. All authors participated in editing the manuscript and approved the final version.

## Funding support

This work is the result of NIH funding, in whole or in part, and is subject to the NIH Public Access Policy. Through acceptance of this federal funding, the NIH has been given a right to make the work publicly available in PubMed Central.

- NIH grants HL133049 and HL139489.
- National Center for Advancing Translational Sciences, National Institutes of Health, through Grant Number UL1TR001873.

## Supplementary Material

Supplemental data

ICMJE disclosure forms

Supporting data values

## Figures and Tables

**Table 1:**
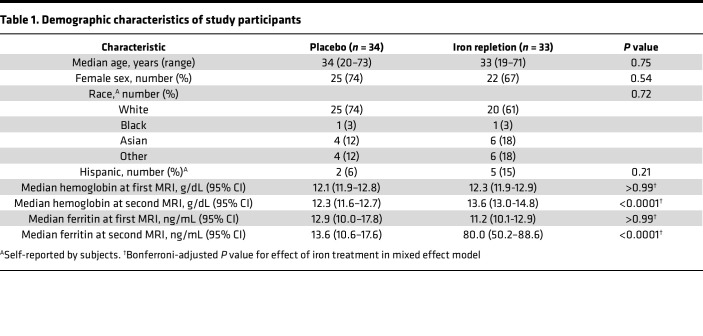
Demographic characteristics of study participants

## Version 1 10/21/2025

In-Press Preview

## Version 2 12/08/2025

Electronic publication
