# Supplementary material for: Effects of iron repletion on brain iron content, myelination, neural network activation, and cognition
Source: JCI Insight. 2025 Oct 21;10(23):e194442. doi: 10.1172/jci.insight.194442 (PMC12890518; doi:10.1172/jci.insight.194442)
Supplement: Supplemental data [file jciinsight-10-194442-s189.pdf]

Table of Contents

Supplementary Methods

Tasks assigned during fMRI scanning Page 2

fMRI Analysis Pipeline and Calculation of Pattern Scores Page 3

QSM Analysis Pipeline Page 4

Supplementary Tables

Supp. Table 1. MRI pulse sequence parameters Page 5

Supplementary Figures

Supp. Figure 1. Cognitive performance scores from NIH Toolbox before and after randomization page 6

Supp. Figure 2. Accuracy on speed and memory tasks performed during functional magnetic resonance imaging (fMRI) before and after randomization page 8

Supp. Figure 3. Relationship between blood laboratory measures of iron status and cognitive scores page 10

Supp. Figure 4. Representative QSM imaging example page 11

References page 12

Supplementary Methods

Tasks assigned during fMRI scanning:

Digit Symbol task:
In the Digit Symbol task (1), a code table is presented on the top of the screen, consisting of numbers 1 through 9, each paired with an associated symbol. Below the code table, an individual number/symbol pair is shown. Subjects are asked to indicate whether each of 90 individual pairs is the same as that in the code table using a differential button press. Subjects are instructed to respond as quickly and accurately as possible.

Letter Comparison task:
In the Letter Comparison task (1), two letter strings, each consisting of 3-5 letters, are presented alongside one another. Subjects indicate whether the strings are the same or different.

Pattern Comparison task:
In the Pattern Comparison task (1), two figures consisting of varying numbers of lines connecting at different angles are presented alongside each other. Subjects indicate whether the figures are the same or different.

Logical Memory task:
In the Logical Memory task (1), subjects read a story and then answer detailed multiple-choice questions about the story, with four possible answer choices. Responses consist of pressing one of four buttons corresponding to the chosen answer.

Word Order Recognition task:
In the Word Order Recognition task (1), a list of 12 words is presented one at a time on the screen, and subjects are instructed to remember the order in which the words are presented. They are then given a probe word at the top of the screen, and four additional word choices below. They are instructed to select the word that *immediately* followed the word given above. The task has two word lists, with ten questions following each list.

Paired Associates task:
In the Paired Associates task (1), six pairs of words are presented, one at a time, on the screen, and subjects are instructed to remember the pairs. Following the pairs, they are given a probe word at the top of the screen and four additional word choices below. They are asked to choose the word that was originally paired with the probe word. The task contains two lists of pairs, with six probe questions for each list. When the proportion of correct trials is calculated for a task, trials where the participant did not respond (i.e., timed out) are not considered in the calculation.

fMRI Analysis Pipeline and Calculation of Pattern Scores

All functional images were realigned to the first volume, corrected for the order of slice acquisition, smoothed with a 5 mm^3 non-linear kernel followed by intensity normalization, and high-pass filtered using a Gaussian kernel and cut-off frequency of 0.008 Hz. To ensure the best alignment of longitudinal scans within each participant, and to avoid any bias towards any one time point, T1-weighted high-resolution anatomic images from both time points were separately co-registered to the respective template for each participant generated as part of FreeSurfer's longitudinal processing stream (2). The first functional volume at each time point was co-registered to the template-aligned T1-weighted image for each respective timepoint using FMRIB Software Library v5.0 (FSL) linear image registration tool (FLIRT) with the normalized mutual information cost function. These transformation parameters were then used to transfer the statistical parametric maps of the subject level analysis to standard space.

The fMRI time-series data was pre-whitened to correct for intrinsic autocorrelations in the data. The FEAT module in FSL was used for subject-level analysis. For each task, we began with block-based analysis. The predictor variables comprising the first level design matrix are composed of epochs representing each unique experimental task relative to rest (i.e., no-performance, fixation periods). Each epoch was convolved with a model of the hemodynamic response function.

Neural network activation pattern scores for the three processing-speed and the three memory tasks were calculated by using the covariance patterns (3) that were optimized to (i) be unique to each cognitive domain and (ii) to predict task performance. For any task activation map $\mathbf{y}$ and activation pattern $\mathbf{v}_j$ (j=1…4), the pattern score is computed as an inner product

$$w_j = \mathbf{y}' \mathbf{v}_j$$

where each voxel loading of the pattern is multiplied by the corresponding voxel value in the activation map, and all products are summed to compute the scalar pattern score. This computation necessitates that the pattern and the activation map are resliced into the same voxel space.

QSM Analysis Pipeline

A 3D multiecho spoiled gradient echo (SPGR) product sequence was used for QSM data acquisition. The data acquisition parameters for all acquisitions were similar across different scanners (Philips Achieva and GE Signa): field of view (FOV) = 24 cm, partial FOV factor = 0.8, acquisition matrix size = 400 x 400 x 80 (Philips) or 512 x 512 x 60 (GE), number of averages = 1, flip angle = 20°, slice thickness = 2 mm, TR = 45-65 msec, number of echoes = 7, first TE = 4.5-6 msec, echo spacing = 5.4-9.1 msec. Parallel imaging was used to accelerate the QSM data acquisition on both GE (ASSET factor = 2) and Philips (SENSE factor = 2) scanners, resulting in a scan time of ~10-13 min. An 8-channel high-resolution brain coil (SENSE-Head-8, Philips) and a 48-channel head coil (GE), were used on the Philips and GE scanners, respectively. Subject heads were positioned in the neutral position.

The acquired complex GRE data was used to estimate the distribution of the susceptibility sources. All computations were performed in Matlab (Mathworks, Natick, MA, USA) using MEDI Toolbox. First, the multi-echo phase data was fitted to a nonlinear model to estimate the frequency maps. Then, the result was spatially unwrapped, after which a background field was removed using Projection onto Dipole Fields (PDF) (4). R_2^* maps were estimated using Auto-Regression on Linear Operations (ARLO)(5). Source-separated susceptibilities were calculated according to the biophysical modeling in the following minimization problem (6, 7):

$$\chi^{+^*}, \chi^{-^*} = \underset{\chi^+,\chi^-}{\operatorname{argmin}} \begin{matrix} \left\| w_1\left(R_2^* - (r^+|\chi^+| + r^-|\chi^-|)\right)\right\|_2^2 + \left\| w_2\left(f - d * (\chi^+ + \chi^-)\right)\right\|_2^2 \\ +2\lambda_1\left|M_{mag}\nabla(\chi^+ + \chi^-)\right|_1 + \lambda_1|M_r\nabla\chi^+|_1 + \lambda_1|M_r\nabla\chi^-|_1 \end{matrix}$$

Here r^+ and r^- are relaxometric constants assumed to be equal to 274 Hz/ppm, f is the tissue field, λ_1 is the regularization parameter, ∇ is a gradient operator, M_{mag} is a binary edge mask derived from the magnitude image, M_r is a binary edge mask derived from R_2^*. The data weight w_2 reflects the reliability of the estimated frequency of each voxel, while w_1 reduces the effects of unreliable R_2^* estimations. Susceptibility values violating physical constraints ($\chi^+ > 0$ and $\chi^- < 0$) were reset to zero. Susceptibility values were expressed in parts per billion (ppb) and have been referenced to cerebrospinal fluid (CSF), defined by R2* < 5 Hz, which was used as the zero reference (8).

T1w images were used for image segmentation using Freesurfer software (9). The resulting segmentations were aligned with the susceptibility images by co-registering the T1w to the GRE magnitude images using the FLIRT algorithm, which is part of the FSL software package (10).

Supplementary Table 1. MRI pulse sequence parameters

Philips Achieva Scanner parameters

Sequence	TE/TR (ms)	Flip Angle (deg)	Matrix (voxels)	Field of View (cm)	Acquisition time (min.)	Slice thick ness/gap (mm)	Slices
**MPRAGE**	2.97/6.57	8	256x256	24x24	~4'55''	1/0	165
**EPI**	20/2000	72	112x112	24x24	~6''/task	3/0	41
**Multi-echo Gradient Echo (QSM)**	5.8/66.7	20	400x400	24x18	~13'32"	2/0	80

GE Signa Scanner parameters

Sequence	TE/TR (ms)	Flip Angle (deg)	Matrix (voxels)	Field of View (cm)	Acquisition time (min.)	Slice thick ness/gap (mm)	Slices
**MPRAGE**	2.96/2202.66	8	256x256	25.6x25.6	~5'	1/0	176
**EPI**	30/1000	72	110x110	22 x22	~6''/task	2/0	64
**Multi-echo Gradient Echo (QSM)**	4.72/42.192	20	512x512	~22x22	~6'30"	2/0	56

Supplementary Fig. 1. Cognitive performance scores from NIH Toolbox before and after randomization. Median standardized score on a task performed using the NIH Toolbox is shown by treatment group from pre- to post-randomization. The vertical bars denote interquartile range. Placebo in blue and iron repletion group in red with colored p-value representing the adjusted p-value from a Šídák's multiple comparisons test of the within group difference from pre- to post-randomization score in a mixed model analysis. P-value in black represents the significance of the mixed model interaction term between treatment group and time point (i.e., is the change different between the groups). Additional fixed effects include the interaction between sex and age. The subject was included in the model as a random effect. An unstructured covariance matrix was used to model the within-patient variance-covariance errors. Pre-specified outcomes were NIH Toolbox standardized scores on (A) Cognition Fluid Composite, (B) Dimensional Change Card Sort, (C) Picture Sequence Memory, (D) List Sorting Working Memory, (E) Inhibitory Control and Attention, (F) Pattern Comparison Processing Speed, (G) Auditory Verbal Learning (Rey), and (H) Auditory Verbal Learning (Rey) Delayed tests. Standard scores have a mean of 100 and standard deviation of 15, with higher scores representing better cognitive performance.

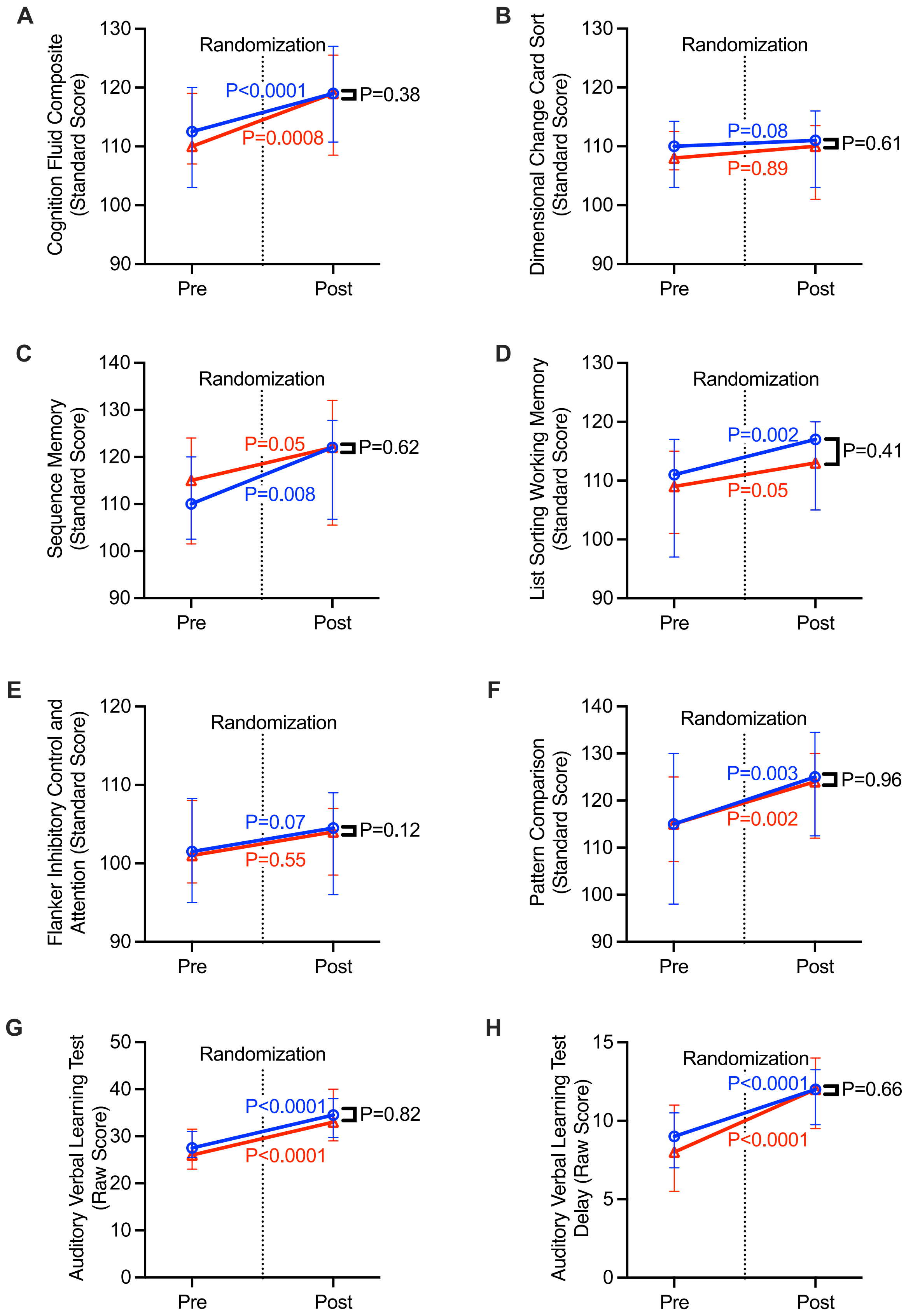
A
Cognition Fluid Composite (Standard Score)
Randomization
P<0.0001
P=0.0008
P=0.38
Pre
Post
B
Dimensional Change Card Sort (Standard Score)
Randomization
P=0.08
P=0.89
P=0.61
Pre
Post
C
Sequence Memory (Standard Score)
Randomization
P=0.05
P=0.008
P=0.62
Pre
Post
D
List Sorting Working Memory (Standard Score)
Randomization
P=0.002
P=0.05
P=0.41
Pre
Post
E
Flanker Inhibitory Control and Attention (Standard Score)
Randomization
P=0.07
P=0.55
P=0.12
Pre
Post
F
Pattern Comparison (Standard Score)
Randomization
P=0.003
P=0.002
P=0.96
Pre
Post
G
Auditory Verbal Learning Test (Raw Score)
Randomization
P<0.0001
P<0.0001
P=0.82
Pre
Post
H
Auditory Verbal Learning Test Delay (Raw Score)
Randomization
P<0.0001
P<0.0001
P=0.66
Pre
Post

Supplementary Fig. 2. Accuracy on speed and memory tasks performed during functional magnetic resonance imaging (fMRI) before and after randomization. Median accuracy on a task performed during fMRI is shown by treatment group from pre- to post-randomization. The vertical bars denote interquartile range. Placebo in blue and iron repletion group in red with colored p-value representing the adjusted p-value from a Šídák's multiple comparisons test of the within group difference from pre- to post-randomization score in a mixed model analysis. P-value in black represents the significance of the mixed model interaction term between treatment group and time point. Additional fixed effects include the interaction between sex and age. The subject was included in the model as a random effect. An unstructured covariance matrix was used to model the within-patient variance-covariance errors. Pre-specified outcomes were accuracy on (A) Letter Comparison, (B) Pattern Comparison, (C) Digit Symbol, (D) Logical Memory, (E) Word Order, and (F) Paired Associates tests.

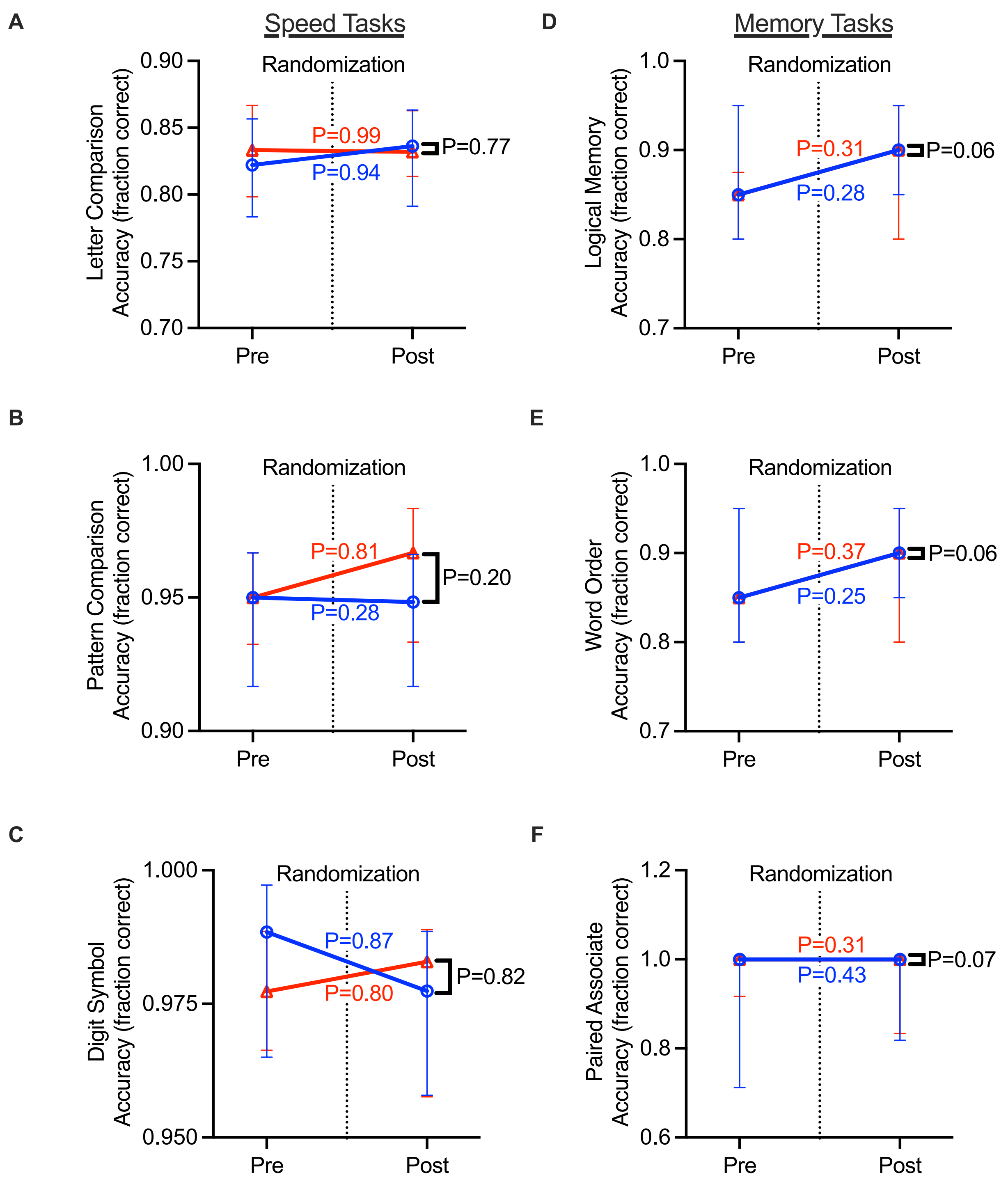
A
Speed Tasks
Randomization
Letter Comparison Accuracy (fraction correct)
0.90
0.85
0.80
0.75
0.70
P=0.99
P=0.94
P=0.77
Pre
Post
B
Randomization
Pattern Comparison Accuracy (fraction correct)
1.00
0.95
0.90
P=0.81
P=0.28
P=0.20
Pre
Post
C
Randomization
Digit Symbol Accuracy (fraction correct)
1.000
0.975
0.950
P=0.87
P=0.80
P=0.82
Pre
Post
D
Memory Tasks
Randomization
Logical Memory Accuracy (fraction correct)
1.0
0.9
0.8
0.7
P=0.31
P=0.28
P=0.06
Pre
Post
E
Randomization
Word Order Accuracy (fraction correct)
1.0
0.9
0.8
0.7
P=0.37
P=0.25
P=0.06
Pre
Post
F
Randomization
Paired Associate Accuracy (fraction correct)
1.2
1.0
0.8
0.6
P=0.31
P=0.43
P=0.07
Pre
Post

Supplementary Fig. 3. Relationship between blood laboratory measures of iron status and cognitive scores. Hemoglobin, ferritin, soluble transferrin receptor, and hepcidin levels were measured in all subjects before and after randomization to iron repletion. In separate mixed effect models for each laboratory measure, which accounted for the repeated measures and the timepoint, the parameter estimate (β) and 95% CI is presented in a Forest plot for each cognitive test performed using the NIH Toolbox standardized scores (as labeled). Additional fixed effects include sex, age, and the interaction between sex and age. The subject was included in the model as a random effect. A variance component covariance matrix was used to model the within-patient variance-covariance errors. AVLT = Auditory Verbal Learning Test (Rey).

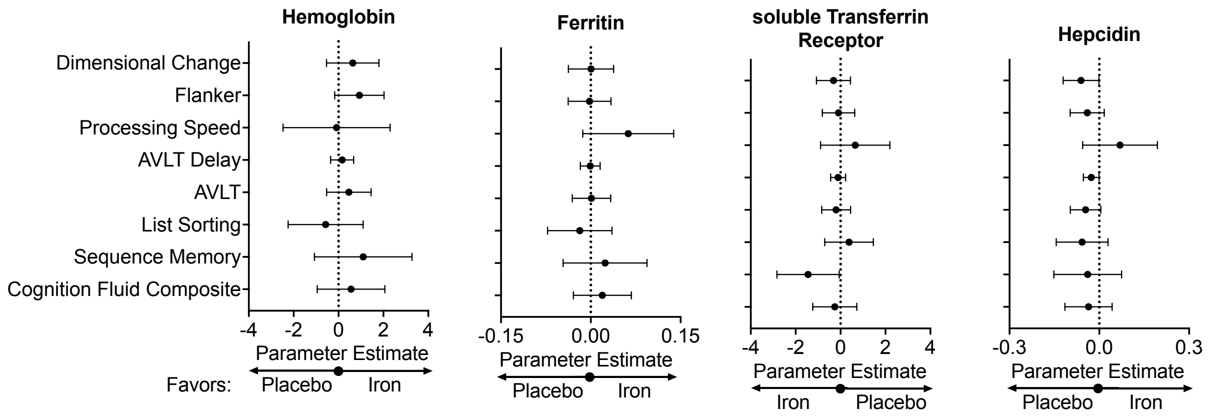

Supplementary Figure 4. Representative QSM imaging example. Flowchart of steps in image analysis along with representative susceptibility, R2*, chi⁺, and chi⁻ maps, as labeled.

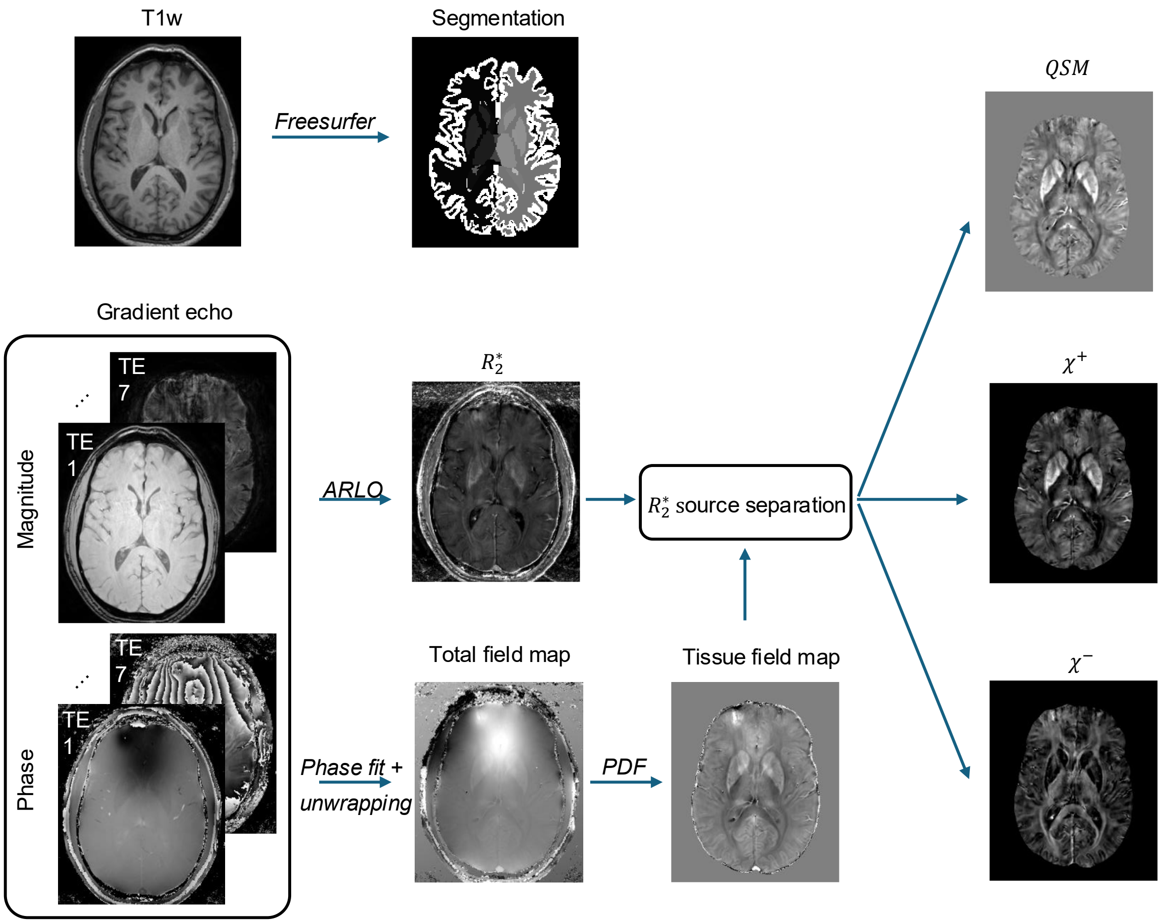

References

1. Stern Y, et al. The Reference Ability Neural Network Study: motivation, design, and initial feasibility analyses. *Neuroimage.* 2014;103:139-51.
2. Reuter M, et al. Within-subject template estimation for unbiased longitudinal image analysis. *Neuroimage.* 2012;61(4):1402-18.
3. Habeck C, et al. Reference ability neural networks and behavioral performance across the adult life span. *Neuroimage.* 2018;172:51-63.
4. Liu T, et al. A novel background field removal method for MRI using projection onto dipole fields (PDF). *NMR Biomed.* 2011;24(9):1129-36.
5. Pei M, et al. Algorithm for fast monoexponential fitting based on Auto-Regression on Linear Operations (ARLO) of data. *Magn Reson Med.* 2015;73(2):843-50.
6. Shin HG, et al. chi-separation: Magnetic susceptibility source separation toward iron and myelin mapping in the brain. *Neuroimage.* 2021;240:118371.
7. Dimov AV, et al. Magnetic Susceptibility Source Separation Solely from Gradient Echo Data: Histological Validation. *Tomography.* 2022;8(3):1544-51.
8. Liu Z, et al. MEDI+0: Morphology enabled dipole inversion with automatic uniform cerebrospinal fluid zero reference for quantitative susceptibility mapping. *Magn Reson Med.* 2018;79(5):2795-803.
9. Fischl B. FreeSurfer. *Neuroimage.* 2012;62(2):774-81.
10. Jenkinson M, et al. Fsl. *Neuroimage.* 2012;62(2):782-90.
